# Comprehensive transcriptome analysis reveals genes in response to water deficit in the leaves of *Saccharum narenga* (Nees ex Steud.) hack

**DOI:** 10.1186/s12870-018-1428-9

**Published:** 2018-10-20

**Authors:** Xihui Liu, Ronghua Zhang, Huiping Ou, Yiyun Gui, Jinju Wei, Hui Zhou, Hongwei Tan, Yangrui Li

**Affiliations:** 1 Key Laboratory of Sugarcane Biotechnology and Genetic Improvement (Guangxi), Guangxi Key Laboratory of Sugarcane Genetic Improvement, Ministry of Agriculture, Sugarcane Research Center, Chinese Academy of Agricultural Sciences, Sugarcane Research Institute, Guangxi Academy of Agricultural Sciences, Nanning, 530007 China; 2 State Key Laboratory for Conservation and Utilization of Subtropical Agro-bioresources, Agricultural College, Guangxi University, Nanning, 530005 China

**Keywords:** Water deficit, Drought, Sugarcane, Leaves, Transcriptome

## Abstract

**Background:**

Sugarcane is an important sugar and energy crop that is widely planted in the world. Among the environmental stresses, the water-deficit stress is the most limiting to plant productivity. Some groups have used PCR-based and microarray technologies to investigate the gene expression changes of multiple sugarcane cultivars under water stress. Our knowledge about sugarcane genes in response to water deficit is still poor.

**Results:**

A wild sugarcane type, *Saccharum narenga*, was selected and treated with drought stress for 22 days. Leaves from drought treated (DTS) and control (CK) plants were obtained for deep sequencing. Paired-end sequencing enabled us to assemble 104,644 genes (N50 = 1605 bp), of which 38,721 were aligned to other databases, such as UniProt, NR, GO, KEGG and Pfam. Single-end and paired-end sequencing identified 30,297 genes (> 5 TPM) in all samples. Compared to CK, 3389 differentially expressed genes (DEGs) were identified in DTS samples, comprising 1772 up-regulated and 1617 down-regulated genes. Functional analysis showed that the DEGs were involved in biological pathways like response to blue light, metabolic pathways and plant hormone signal transduction. We further observed the expression patterns of several important gene families, including aquaporins, late embryogenesis abundant proteins, auxin related proteins, transcription factors (TFs), heat shock proteins, light harvesting chlorophyll a-b binding proteins, disease resistance proteins, and ribosomal proteins. Interestingly, the regulation of genes varied among different subfamilies of aquaporin and ribosomal proteins. In addition, DIVARICATA and heat stress TFs were first reported in sugarcane leaves in response to water deficit. Further, we showed potential miRNAs that might be involved in the regulation of gene changes in sugarcane leaves under the water-deficit stress.

**Conclusions:**

This is the first transcriptome study of *Saccharum narenga* and the assembled genes are a valuable resource for future research. Our findings will improve the understanding of the mechanism of gene regulation in sugarcane leaves under the water-deficit stress. The output of this study will also contribute to the sugarcane breeding program.

**Electronic supplementary material:**

The online version of this article (10.1186/s12870-018-1428-9) contains supplementary material, which is available to authorized users.

## Background

Sugarcane is a C_4_ grass that belongs to the family Poaceae, sub-family Panicoideae and tribe Andropogoneae. It is an important industrial crop widely grown in tropical and subtropical areas, due to the highly yield of sugar [1]. However, the production of sugarcane is always affected by the environmental stresses, such as water, cold and salt. Among these conditions water stress is the most limiting to plant productivity [1].

Water stress always induces a number of biochemical and physiological responses in plants, such as stomatal closure, repression of cell growth and photosynthesis, and activation of respiration [2]. Studies have been demonstrated to understand the gene expression changes under the water-deficit stress in several model species, such as Arabidopsis [3, 4], maize [5], rice [6], tomato [7], banana [8], soybean [9], and other plants [10–13]. They have shown that abscisic acid (ABA)-dependent and ABA-independent regulatory systems are two major pathways for plants to defense against the water-deficit stress [14]. According to their functions, genes in response to water deficit can be divided into two groups. The first group of gene products are involved in the protection of cells and in the regulation of signal transduction pathways of stress responses, such as chaperons, late embryogenesis abundant (LEA) proteins, water channel proteins, heat shock proteins and lipid-transfer proteins. The second group of gene products in response to water deficit in plants is comprised of regulatory proteins that are involved in further regulation of signal transduction and stress-responsive gene expression, such as various TFs and dehydration-responsive elements [2, 14, 15].

Some studies have used PCR and microarray technologies to investigate the gene expression profiles of different sugarcane cultivars under water stress. For example, Rodrigues and colleagues identified 91 genes up-regulated in both tolerant (SP83–5073) and sensitive (SP90–1638) sugarcane cultivars, such as heat shock protein 17.2, resistance protein LR10 and transcription factor E2Fe [1]. Carolina and colleagues identified 928 sense transcripts and 59 antisense transcripts differentially expressed in the aerial parts of sugarcane (SP90–1638) submitted to drought for 24, 72 and 120 h [16]. Another study by Rodrigues and colleagues has shown that 1670 genes were differentially expressed in sugarcane (SP83–2847) under mild, moderate and severe water deficit stresses [17]. Gupta and colleagues have identified 25 clusters (EST groups) induced by water-deficit stress in sugarcane (CoS 767) [18]. Iskandar and colleagues have tested 51 genes in the culms of multiple sugarcane cultivars and reported water-deficit stress related genes including genes encoding enzymes involved in amino acid metabolism, a sugar transporter and a transcription factor [11]. Prabu and colleagues have investigated the gene expression profiles of sugarcane (Co740) under varied levels of water deficiency stress using PCR-based cDNA suppression subtractive hybridization technique and have found 158 clones up-regulated under water-deficit stress, which mainly function in cellular organization, protein metabolism, signal transduction, and transcription [19]. However, our knowledge of the gene expression profiles and gene regulation in sugarcane leaves under the water-deficit stress is still poor.

*Saccharum narenga* (Nees ex Steud.) Hack, also named as *Narenga porphyrocoma* (Hance) Bor, is a wild species of sugarcane distributed mainly in Asia-temperate (China and eastern Asia) and Asia-tropical (India and Indo-China) areas. This species has a smaller genome size (2n = 30) and many excellent characters, such as drought tolerance, precocity, stocky stem, high tillering ability, red rot tolerance, smut tolerance and mosaic disease resistance. These advantages enable it to be used in sugarcane breeding program. To understand the gene expression profiles of this species under the water-deficit stress, both paired-end and single-end sequencing technologies were used to sequence the cDNA libraries of drought treated leaves and control (CK) plants. Differential expression analysis identified some gene products that have been reported to be involved in response to water deficit, such as various transcription factors (TFs), dehydrins, aquaporins, heat shock proteins, ribosomal proteins and auxin-related proteins. We not only identified novel water-deficit related genes in sugarcane leaves, such as light harvesting chlorophyll a-b binding protein and multiple TFs, but also found, for the first time, that the regulation of genes differ across aquaporin and ribosomal protein subfamilies. Our results will contribute to understand the drought tolerance mechanism in sugarcane and will contribute to the field of sugarcane breeding program. This is the first transcriptome of *Saccharum narenga* species, so the gene sequences can be referred by future studies.

## Methods

### Plant material and drought treatment

The *Saccharum narenga* plants were collected from a barren mountain not far from Nanning of China (22°53′06.7″N 108°21′36.6″E). They were proved to be Guangxi Hebawang NO. 1, which is from the clonal *Saccharum narenga*. Six sugarcane plantlets were transplanted in a pod filled with a mixture of peat soil, washed sand, vermiculite and perlite (total weight: ~ 17.5 kg). Then, ~ 20 pods were transferred and maintained in a greenhouse of Guangxi Academy of Agricultural Sciences on 2nd May 2014. The greenhouse is equipped with a system for monitoring temperature and relative humidity (mean temperature: 29° ± 4 °C, mean relative humidity: 75 ± 5%). Every day the plants were watered sufficiently (~ 800 mL per pod). After 6 months, three pods (DTS-R1, DTS-R2 and DTS-R3) were randomly selected for drought treatment while another three (CK-R1, CK-R2 and CK-R3) pods which have similar growing plants were used as CK. Drought treatment was performed by withholding water for 22 days. One leaf from each pod was randomly selected, immediately stored in liquid nitrogen and stored at − 80 °C before RNA isolation.

### Total RNA extraction

Total RNA of each leaf was extracted by using TRIzol reagent, as previously described [20, 21]. In brief, 100 mg of leaf sample were mixed with 1 mL TRIzol reagent, homogenized using a power homogenizer and centrifuged at 12000×g for 10 min at 4 °C. Then, the fatty layer was removed and discarded. The supernatant was transferred into a new tube and added with chloroform (0.2 mL). After shaking for 15 s, the tube was incubated at room temperature for 3 min and centrifuged at 12000×g for 15 min at 4 °C. The aqueous phase was moved into a new tube and added with RNase-free glycogen (10 μg) and 100% isopropanol (0.5 mL), followed by an incubation at room temperature for 10 min. Then, the tube was centrifuged at 12000×g for 10 min at 4 °C and the pellet was transferred into a new tube with 75% ethanol (1 mL). Then, the tube was vortexed gently and centrifuged at 7500×g for 5 min at 4 °C. The RNA pellet was air-dried, suspended in RNase-free water and water bathed at 60 °C for 10 min. An Agilent 2100 Bioanalyzer was used to evaluate the quantity and quality of the total RNA of each sample.

### Transcriptome library construction and sequencing

We used both paired-end and single-end strategies for transcriptome sequencing. The cDNA library of each sample was constructed using the TruSeq RNA Sample Preparation Kit v2 (Illumina) and sequenced on the Illumina HiSeq 2000 platform, according to protocols. Briefly, equal amount (20 μg) of total RNA (RIN > 8.0) of each sample was used to enrich the mRNAs using the Dynal Oligo(dT) beads (Invitrogen). The mRNAs were then chemically fragmented into ~ 200 nt fragments using divalent cations (Elute/Prime/Fragment Mix buffer, Illumina) under elevated temperature, followed by the cDNA synthesis. The cDNA fragments were end-repaired using End Repair Mix (Illumina) and purified. Then, we used TruSeq Paired-End Cluster Kit v3 (Illumina PE-401-3001) and TruSeq SBS HS Kit v3 (Illumina FC-401-3001) to generate the final cDNA libraries for paired-end (90 bp × 2) and single-end (50 bp) sequencing, respectively, according to the protocols [22]. Mixture of CK samples was named as CK-MIX and mixture of DTS samples was named as DTS-MIX. DTS-MIX and CK-MIX were processed with paired-sequencing while DTS-R1, DTS-R2, DTS-R3, CK-R1, CK-R2 and CK-R3 were processed with single-end sequencing.

### De novo analysis and transcriptome annotation

Raw reads from the paired-end sequencing were cleaned by removing low quality reads and reads with adaptors or ambiguous base ‘N’. The resulted high-quality reads were then quality controlled using FASTQC ((http://www.bioinformatics.babraham.ac.uk/projects/fastqc/). Trinity software (v2.4.0) was used for the de novo transcriptome analysis with default parameters [23]. Then, using the superTranscripts function provided by Trinity we constructed the possible ‘gene’ sequences of sugarcane [24].

To perform the transcriptome annotation, we first identified likely coding sequences in the sugarcane transcriptome using TransDecoder (https://transdecoder.github.io/) under default parameters. Then, Trinotate (v2.0.2, available at http://trinotate.github.io/) was used to annotate the deduced proteins. Briefly, the sugarcane deduced proteins were searched against the UniProtKB/Swiss-Prot database to identify known protein sequences. HMMER was used to predict functional domains of the deduced proteins by mapping them to the PFAM database [25]. Next, SignalP [26], RNAMMER [27] and TMHMM Sever 2.0 [28] were used to annotate potential signal peptides, ribosomal RNA transcripts and transmembrane domains, respectively, for the assembled transcriptome. The deduced proteins were also searched against the EggNOG database (v 4.1, http://eggnogdb.embl.de/) to identify known proteins in EuKaryotic Orthologous Groups (KOG), Clusters of Orthologous Groups (COGs), and non-supervised orthologous groups (NOGs) [29]. All the above annotations were loaded to a Trinotate SQLite database and a final annotation was produced. The cut-off of e-values for best hits was set to 1e-5.

### Gene ontology and KEGG pathway annotation

We also annotated the assembled genes related to Gene Ontology (GO) and Kyoto Encyclopedia of Genes and Genomes (KEGG) pathway. In brief, all the assembled genes were search against NCBI non-redundant (NR), UniProt and KEGG pathway databases using the BLAST software [30]. Cut-offs and other filters were applied to select the best hits, as previously described [12].

### Reads alignment and gene expression profiling

The Trinity transcripts were quantified using the RSEM (RNA-Seq by Expectation-Maximization) method [31]. After data cleaning described above, both paired-end and single-end sequencing reads were aligned to the assembled transcriptome using Bowtie2 [32]. Then, RSEM tool was used to identify the gene expression levels in all samples [31]. In this study, TPM (transcripts per million reads) method was used for normalization and lowly expressed genes (< 5 TPM) were filtered. Recommended parameters by Trinity were used for Bowtie2 and RSEM.

### Differential expression analysis

Differentially expressed genes in DTS samples relative to CK were identified using the edgeR software [33]. Following parameters were used to select the differentially expressed genes: i) >  5 TPM in at least one sample, ii) Log2FC (log2 fold change) > 1 (up-regulated) or Log2FC < − 1 (down-regulated), iii) *p*-value < 0.5 and iv) FDR (false discovery rate) < 0.05.

### Functional analysis

Enrichment of GO terms and KEGG pathways was analyzed to predict the functions of candidate genes. Fisher’s exact test was used to calculate the *p-value* which represents the significance of enrichment, and an R package named ‘q-value’ was used to correct the *p-value* for each GO term/ KEGG pathway and control the false discovery rate. Significant GO terms and KEGG pathways were selected if the *p-value* < 0.05 and *q-value* < 0.05. GO terms and KEGG pathways not related to plant bio activities were filtered.

### qRT-PCR

Quantitative real-time PCR (qRT-PCR) experiment was performed to confirm the expression patterns in the leaves, following the protocol [12]. In brief, the total RNA was extracted using TRIzol reagent (Invitrogen) and quality-controlled using the Agilent 2100 Bioanalyzer. Primer3 (http://bioinfo.ut.ee/primer3-0.4.0/) was used to predict the forward and reverse primers for 9 randomly selected genes and the endogenous control (actin). All the primers were synthesized at BGI-Shenzhen. The cDNA synthesis and qRT-PCR experiments were performed, as previously described [12]. Three reactions were conducted for each candidate gene in every sample. Then, the average Ct (cycle threshold) was calculated and ΔCt was used to show the expression level of each candidate gene (relative to actin). ΔΔCt method was used to show the different expression of a gene in drought-stressed sugarcane leaves, compared to CK, as described [12].

### miRNA target prediction

miRNA target prediction was performed using the plant miRNA target prediction software psRobot with default parameters [34].

## Results

### De novo analysis of *Saccharum narenga* leaf transcriptome

**Fig. 1:**
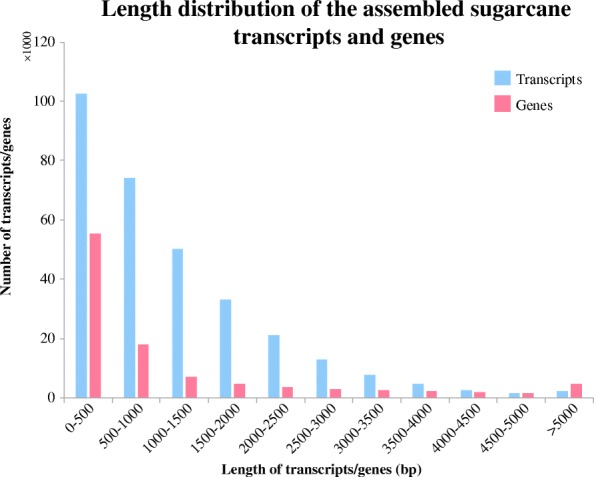
Length distribution of the assembled sugarcane transcripts and genes

Six *Saccharum narenga* plants were obtained from a barren mountain in Guangxi, China and transplanted into a greenhouse of Guangxi Academy of Agricultural Sciences. During the maturity period, three of them (DTS) were not watered for 22 days and the rest (CK) were treated with sufficient water. One leaf of each plant was randomly selected for total RNA isolation and subsequent transcriptome sequencing. Two paired-end (2 × 90 bp) libraries (CK-MIX and DTS-MIX) and six single-end (50 bp) libraries for CK (CK-R1, CD-R2 and CK-R3) and DTS (DTS-R1, DTS-R2 and DTS-R3) samples were sequenced on the Illumina HiSeq 2000 platform. The paired-end sequencing produced a total of ~ 15.4 G data (~ 171 million raw reads). After data cleaning, we obtained 156.8 million clean reads and processed the de novo analysis using Trinity software (v2.4.0) [23] (Table 1). The assembly produced a total of 312,800 transcripts, which contain 361,982,856 bases (534 M in size) (Table 1). The N50 statistic was 1771, which meant that more than 50% of the transcripts were longer than 1771 bp. Then, identification of the superTranscripts function found that these transcripts were from 104,644 genes that contain 89,330,093 bases (122 M in size) (Table 1). The N50 statistic was 1605 while the average length of all the genes was 853.66 bp (Table 1). The length distribution of all the assembled sugarcane genes was shown in Fig. 1, which indicated that 16.8% of the total transcripts and 18.6% of the total genes were longer than 2000 bp. This is the first time to study the *Saccharum narenga* leaf transcriptome, so it is difficult to evaluate the number of transcripts/genes in *Saccharum narenga* due to the missing information of its genome sequence and annotation.

**Table 1:** Overview of the assembled sugarcane transcriptome

Type	CK_MIX	DTS_MIX
Total paired-end reads	79,172,916	77,639,556
Total trinity transcripts	312,800	
N50 (transcripts)	1771	
Total assembled bases (transcripts)	361,982,856	
Total trinity genes	104,644	
N50 (genes)	1605	
Average length (genes)	853.66	
Total assembled bases (genes)	89,330,093	
GC (%)	47.36

### Annotation of the assembled sugarcane genes

**Fig. 2:**
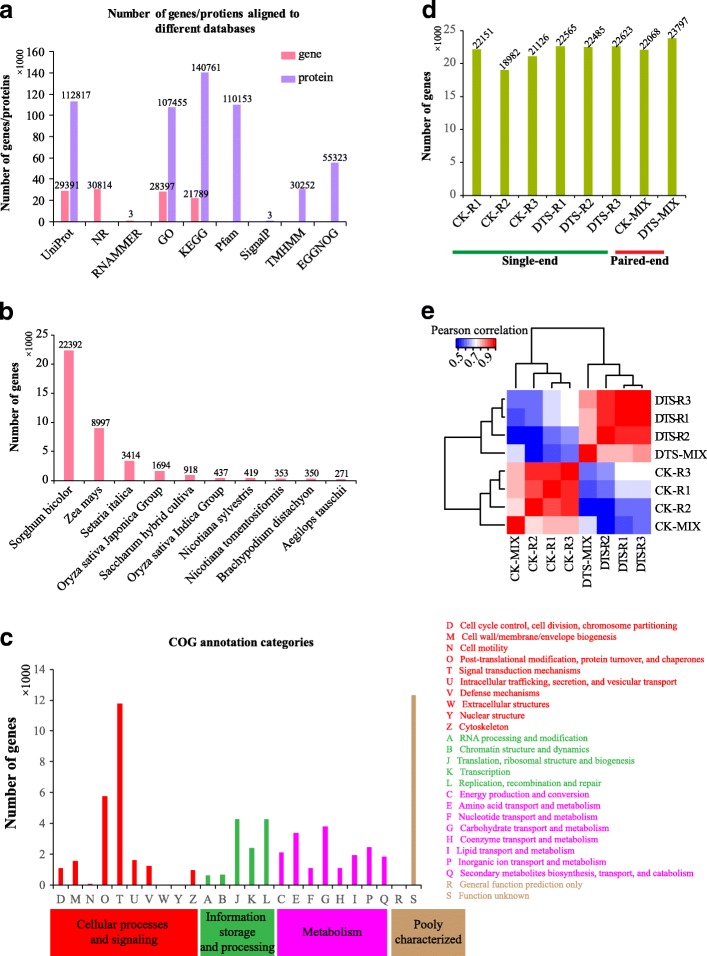
Annotation of the assembled sugarcane transcriptome and gene expression profiling. **a** Number of genes and predicted proteins aligned to different databases. **b** Distribution of species aligned by the assembled sugarcane genes. **c** COG annotation for the assembled sugarcane genes. **d** Number of genes identified in each sample (> 5 TPM). **e** Heat map of sample correlation based on the gene expression profile

We next annotated the assembled sugarcane genes by mapping them to multiple databases, such as NCBI non-redundant (NR), UniProt, GO and KEGG databases. Fig. 2a shows that 29,391 (28.09%), 30,814 (29.45%), 28,397 (27.14%) and 21,789 (20.82%) gene sequences were aligned to the UniProt, NR, GO and KEGG databases, respectively. In addition, 3 rRNA sequences were predicted by RNAMMER [27]. Further, in the NR mapping results we retrieved and counted the genes aligned to different species and the top 10 species aligned by the assembled sugarcane genes were listed in Fig. 2b, which showed that 22,392 genes, 21.40% of the assembled sugarcane genes, were aligned to *Sorghum bicolor*, followed by *Zea mays* (8997 genes)*, Setaria italica* (3414 genes)*, and Oryza sativa Japonica Group* (1694 genes). GO annotation (Additional file 1) revealed that 13,157, 13,920 and 10,363 genes were involved in “cellular process”, “metabolic process” and “catalytic activity”, respectively. In addition, “developmental process”, “growth” and “response to stimulus” were identified to involve 1553, 278 and 3438 genes, respectively. Top five KEGG pathways involved by the sugarcane genes were “metabolic pathways” (ko01100, 4961 genes), “biosynthesis of secondary metabolites” (ko01110, 3267 genes), “plant-pathogen interaction” (ko04626, 1786 genes), “neurotrophin signaling pathway” (ko04722, 1581 genes) and “apoptosis” (ko04210, 1486 genes).

Further, TransDecoder (https://transdecoder.github.io/) was used to identify 192,553 likely proteins encoded by 35,424 of the assembled sugarcane genes (Fig. 2a). It was found that 113,817 (58.59%), 107,455 (55.81%), 140,761 (73.10%) and 110,153 (57.21%) of the deduced proteins were aligned to UniProt, GO, KEGG and Pfam databases, respectively. SignalP [26] identified 3 proteins containing signal peptides and TMHMM [28] identified 30,252 proteins that were highly similar to membrane proteins (Fig. 2a). Then, the deduced proteins were aligned to EggNOG database (v4.1, http://eggnogdb.embl.de). In total, 55,323 deduced proteins were annotated into 1374 COGs, 41 KOGs and 333 NOGs. We found that the most significant known COG category was “signal transduction mechanisms”, which involved 11,748 deduced proteins, followed by “post-translational modification, protein turnover, and chaperones” that involved 5782 deduced proteins (Fig. 2c). Meanwhile, 12,304 deduced proteins (Fig. 2c) were annotated as “function unknown”. Different annotation perspectives of the assembled sugarcane transcriptome will help understand of the process of sugarcane leaves in response to water deficit. In addition, the reasons of some genes without encoding capacity require to be explored with more experiments [12]. In total, 39,716 of the assembled genes were annotated to be similar to other species while 22,509 of them were predicted to encode proteins. This means that 12,915 of the assembled genes were predicted to encode proteins that have not been reported. More experiments are required to study the novel genes/proteins and their functions in *Saccharum narenga* under water stress.

### Gene expression profiling

To profile the gene expression in CK and DTS samples, both paired-end and single-end sequencing reads were aligned to the assembled sugarcane gene sequences using Bowtie2 [32]. Then, RSEM [31] was used to estimate the abundance of each gene in the samples and TPM (transcripts per million reads) method was used for normalization. After lowly expressed genes (< 5 TPM) were filtered, a total of 30,297 genes were identified across all samples (Additional file 2). In detail, 24,598 and 25,584 genes were identified in CK (CK-R1: 22,151, CK-R2: 18,982, CK-R3: 21,126) and DTS (DTS-R1: 22,565; DTS -R2: 22,485; DTS -R3: 22,623) samples, respectively, using single-end sequencing (Fig. 2d). And 22,068 and 23,797 genes were identified in CK-MIX and DTS-MIX, respectively, by paired-end sequencing (Fig. 2d). A heat map (Fig. 2e) showed that the correlation between replicates was high and DTS samples were distinct from CK samples based on both paired-end and single-end sequencing data.

### Differential expression analysis

**Fig. 3:**
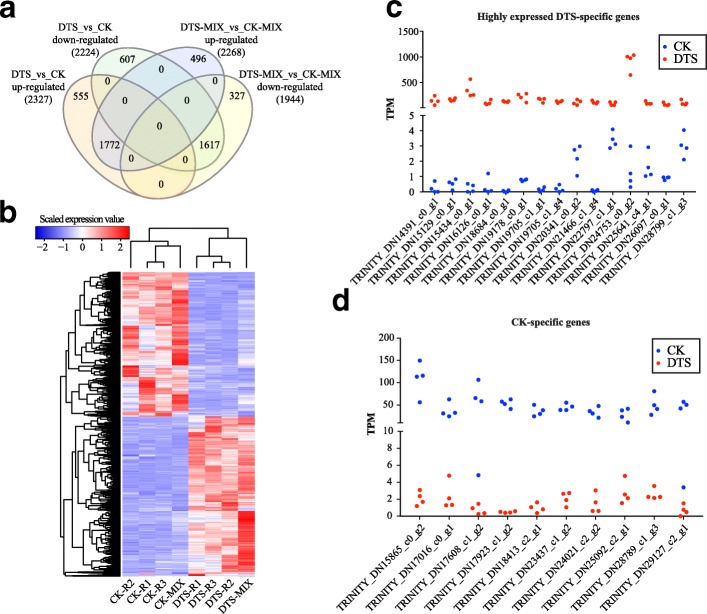
Differential expression analysis. **a** Venn diagram of DEGs identified by paired-end and single-end sequencing technologies. **b** Heat map of the DEG expression levels. **c** Highly expressed genes (> 100 TPM) exclusively identified in DTS samples. **d** Top 10 highly expressed genes identified exclusively in CK samples

We next used edegR [33] to identify differentially expressed genes (DEGs) in sugarcane leaves in response to water deficit. As shown in Fig. 3a, single-end and paired-end sequencing identified 4551 (2327 up-regulated and 2224 down-regulated) and 4212 (2268 up-regulated and 1944 down-regulated) genes, respectively, differentially expressed in DTS samples, compared to CK (Additional file 3). It was found that the expression patterns of 3389 (1772 up-regulated and 1617 down-regulated) genes were consistent in paired-end and single-end sequencing (Fig. 3a, Additional file 4). We also used a heat map to show the expression changes of genes in sugarcane leaves in response to water deficit (Fig. 3b). Among the DTS up-regulated genes 843 were lowly expressed (< 5 TPM) in CK. We showed the highly expressed (> 100 TPM) DTS-specific genes in Fig. 3c, such as TRINITY_DN24753_c0_g2 encoding transposon Tf2–9 polyprotein, TRINITY_DN21466_c1_g1 encoding non-specific lipid-transfer protein 2, TRINITY_DN19178_c0_g1 encoding low molecular mass early light-inducible protein HV60, TRINITY_DN19705_c1_g1 encoding dehydrin DHN1, TRINITY_DN18684_c0_g1 encoding late embryogenesis abundant (LEA) protein and TRINITY_DN14391_c0_g1 encoding low temperature-induced protein lt101.2. In Fig. 3d, we showed top 10 genes (expression ranged from 28.48 TPM to 108.75 TPM) that were identified in CK samples but lowly expressed (< 5 TPM) in DTS samples. Except for the genes only identified in DTS or CK samples, we also identified the differentially expressed genes that encode various protein products, including ABC transporter, auxin response factor, light-regulated protein, chlorophyll a-b binding protein and galactinol-sucrose galactosyltransferase.

### Functional analysis

**Fig. 4:**
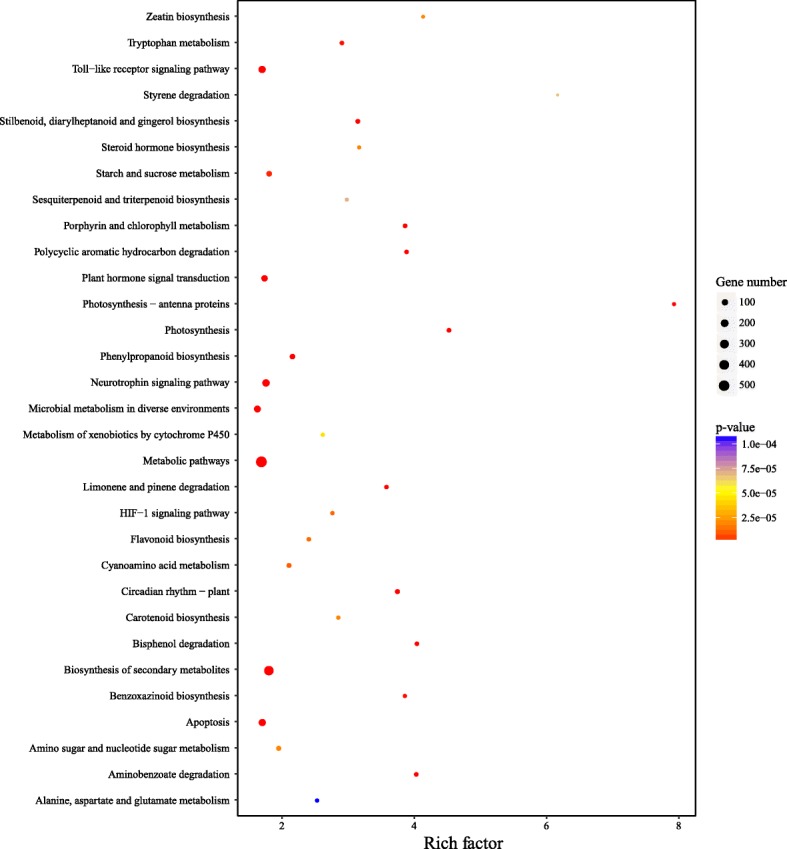
KEGG enrichment analysis of the differentially expressed genes

To gain insights of sugarcane leaf genes in response to water deficit, functional analysis was performed to identify enriched GO terms and KEGG pathways involved by the DEGs. Among the significant GO terms (Table 2), we found that 19 genes were involved in “response to blue light” (GO:0009637), 12 genes were related to “chloroplast part” (GO:0044434) and 9 genes were enriched in “negative regulation of defense response” (GO:0031348). Next, we used a bubble plot to show the significant KEGG pathways involved by the DGEs (Fig. 4). It showed 537 DEGs were involved in “metabolic pathways” (ko01100), which is the most significant. Interestingly, we identified 40 DEGs involved in “circadian rhythm - plant” (ko04712) that is related to environmental adaptation. In addition, 161 and 107 DEGs were involved in the pathways of “apoptosis” (ko04210) and “plant hormone signal transduction” (ko04075), respectively. In addition, plant hormone related pathways “flavonoid biosynthesis” (ko00941), “steroid hormone biosynthesis” (ko00140), “zeatin biosynthesis” (ko00908) and “sesquiterpenoid and triterpenoid biosynthesis” (ko00909) were identified to involve 28, 17, 12 and 16 DEGs, respectively. Although it is not clear about the functions of these pathways in sugarcane leaves in response to water deficit, previous studies have shown that metabolism, auxin, abscisic acid (ABA), cytokinins and ethylene are related with cell growth and cell death in plants [35–37].

**Table 2:** GO enrichment analysis of DEGs in DTS samples compared to CK

Type	ID	Term	Number	Enrich_factor	*p*-value
Biological process	GO:0009886	post-embryonic morphogenesis	1	1.09	5.19E-06
	GO:0015977	carbon fixation	4	3.91	2.57E-05
	GO:0015786	UDP-glucose transport	2	0.75	5.55E-05
	GO:0009695	jasmonic acid biosynthetic process	5	0.50	9.06E-05
	GO:0031348	negative regulation of defense response	9	0.62	0.0001101
	GO:0006014	D-ribose metabolic process	3	1.38	0.0001256
	GO:1902582	single-organism intracellular transport	1	0.72	0.0002646
	GO:0045491	xylan metabolic process	2	1.47	0.0003353
	GO:0009637	response to blue light	19	1.82	0.0003557
	GO:0086010	membrane depolarization during action potential	2	2.79	0.0005124
	GO:0048869	cellular developmental process	4	6.18	0.0005507
	GO:0044723	single-organism carbohydrate metabolic process	5	2.07	0.0005799
	GO:0010236	plastoquinone biosynthetic process	2	1.89	0.000751
	GO:0009750	response to fructose	5	0.45	0.000751
	GO:0046483	heterocycle metabolic process	1	1.13	0.0007543
	GO:0051726	regulation of cell cycle	2	0.28	0.0008238
	GO:0055088	lipid homeostasis	2	0.69	0.0009208
Molecular function	GO:0015254	glycerol channel activity	9	1.89	0.0003135
Cellular component	GO:0009522	photosystem I	19	2.10	0.001842
	GO:0044434	chloroplast part	12	2.35	0.004865

### Sugarcane genes in response to water deficit

We next analyzed the DEGs in different categories that might be related to sugarcane leaves in response to water deficit, such as aquaporin, LEA, auxin related proteins, transcription factors, heat shock proteins, chlorophyll a-b binding proteins and some other proteins. In Table 3, we showed the statistics of these gene families in this study, including the number of genes identified and the number of dysregulated in DTS samples compared to CK.

**Table 3:** Important gene families in response to water deficit in sugarcane leaves

Family	annotated	identified (> 5 TPM)	up-regulated	down-regulated
Aquaporin	60	22	8	3
LEA	13	6	6	0
auxin-related	175	72	7	7
heat shock protein	68	33	5	1
transcription factor	882	425	70	36
chlorophyll a-b binding protein	59	27	0	22
light-regulated proteins	1	1	0	1
non-specific lipid-transfer protein	40	11	5	2
disease resistance protein	1458	456	14	45
ribosomal protein	809	333	2	18
dehyrin	5	4	4	0

#### Aquaporin

**Fig. 5:**
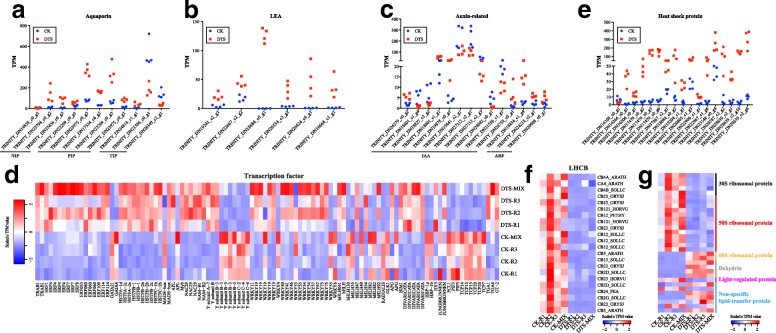
Sugarcane genes in response to the water-deficit stress. We investigated the expression levels of DEGs in several families, such as **a** aquaporin, **b** LEA, **c** auxin-related protein, **d** transcription factor, **e** heat shock protein, **f** light harvest chlorophyll a-b binding protein, and **g** some other protein families

In this study, we identified 60 genes encoding aquaporin (Table 3), of which 11 were differentially expressed (8 up-regulated and 3 down-regulated) in DTS samples relative to CK (Additional file 4). Interestingly, the regulation of genes varied among different aquaporin subfamilies (TIP, NIP and PIP). In general, genes encoding NIPs and PIPs were up-regulated in sugarcane leaves in response to the water-deficit stress while genes encoding TIPs were down-regulated (Fig. 5a).

#### LEA

There were 13 genes encoding LEA in the assembled sugarcane leaf transcriptome (Table 3). We identified 6 LEA genes > 5 TPM in all samples and all of them were up-regulated in the sugarcane leaves in response to water deficit. Up-regulated LEA genes were annotated to have the capacity to encode proteins LEA5D_GOSHI, LEA3_MAIZE, LEA14_GOSHI, LEA5_CITSI and LEA34_GOSHI. It is notable that 4 out of the 6 LEA DEGs were not detected (< 5 TPM) in CK (Fig. 5b).

#### Auxin-related protein

We identified 175 genes encoding auxin-related proteins (Additional file 4), including auxin response factors (ARFs), auxin-responsive proteins (IAAs), auxin efflux carrier components, auxin-repressed proteins, auxin-binding proteins and auxin-induced proteins. However, only 15 of them were differentially expressed in DTS samples compared to CK. They include 2 genes encoding ARFs, 2 genes encoding auxin-induced proteins and 11 genes encoding IAAs. It is interesting that DEGs encoding ARFs and auxin-induced proteins (TRINITY_DN26758_c0_g1 and TRINITY_DN18434_c1_g4) were up-regulated in sugarcane leaves in response to water deficit, however, genes encoding IAAs were down-regulated, generally, in DTS samples compared to CK (Fig. 5c).

#### Transcription factor

There were 882 sugarcane genes that have the capacity of encoding TFs, of which 106 were differentially expressed (Table 3). These 106 TF DEGs include 70 up-regulated genes, which can encode bZIP TF TRAB1, ERFs (ethylene-responsive TFs), heat stress TFs, NAC TFs, WRKY TFs and DIVARICATA TFs, and 36 down-regulated genes, which can encode GATA TFs, Nuclear TF Y subunits, bHLH TFs, WRKY TFs and TCP TFs. A heat map of their expression values in DTS and CK samples were shown in Fig. 5c. ERFs and heat stress TFs were identified to be induced by water deficit in sugarcane leaves while TCP TFs were suppressed. It is difficult to determine the regulation of WRKY TFs in sugarcane leaves in response to water deficit. More experiments are required to explore the functions of WRKY TFs, as well as other TF genes.

#### Heat shock protein

We next analyzed the expression changes of heat shock proteins (HSPs) in sugarcane leaves in response to water deficit. Among the 157 HSP genes, 17 (10.8%) were differentially expressed (16 up-regulated and 1 down-regulated) in DTS samples compared to CK (Table 3, Additional file 4). Compared to the up-regulated HSP genes, the down-regulated HSP gene TRINITY_DN16108_c0_g1 was lowly expressed in both DTS (ranged from 5.71 to 11.24 TPM) and CK (ranged from 2.29 to 3.65 TPM) samples (Fig. 5e). Interestingly, HSP 70 kDa proteins are the largest family encoded by the differentially expressed HSP genes.

#### Light-harvesting chlorophyll a-b binding protein

Genes encoding light-harvesting chlorophyll a-b binding proteins (LHCB) are the highest expressed gene family in the sugarcane leave transcriptome (Additional file 2). In total, 59 LHCB genes were annotated in the assembled sugarcane transcriptome and 22 (37.29%) of them were down-regulated in DTS samples compared to CK (Table 3**,** Fig. 5f). Three LHCB genes were very abundant (> 10,000 TPM) in CK samples, such as TRINITY_DN20351_c2_g1 (CB23_ORYSJ), TRINITY_DN25403_c0_g1 (CB23_ORYSJ), TRINITY_DN27533_c1_g1 (CB2G_SOLLC). Because of the water-deficit stress, they were dropped by ~ 4 times to < 4500 TPM (Additional file 4).

#### Other water-deficit associated genes

We also observed the expression patterns of some other gene families that might relate to the drought tolerance of sugarcane leaves, such as genes encoding light-regulated protein, non-specific lipid-transfer protein, putative disease resistance proteins, dehydrin and ribosomal proteins (Fig. 5g, Additional file 4). The disease resistant protein family might be the largest gene family identified in this study. We identified 1458 genes that can encode disease resistance proteins and 456 were expressed more than 5 TPM. Of them, 14 up-regulated and 45 down-regulated genes were identified in DTS samples compared to CK (Table 3, Additional file 4). It is interesting that genes encoding 30S and 50S ribosomal proteins were up-regulated while genes encoding 60S ribosomal proteins were down-regulated. This indicates they may have diverse functions in sugarcane leaves in response to water deficit. All the 4 genes encoding dehydrin proteins were up-regulated while the only DEG (TRINITY_DN28547_c1_g1) encoding light-regulated protein was down-regulated in DTS samples compared to CK (Additional file 4). Among the DEGs encoding non-specific lipid-transfer proteins, 2 were down-regulated while 5 were up-regulated in DTS samples relative to CK (Additional file 4).

### qRT-PCR

**Fig. 6:**
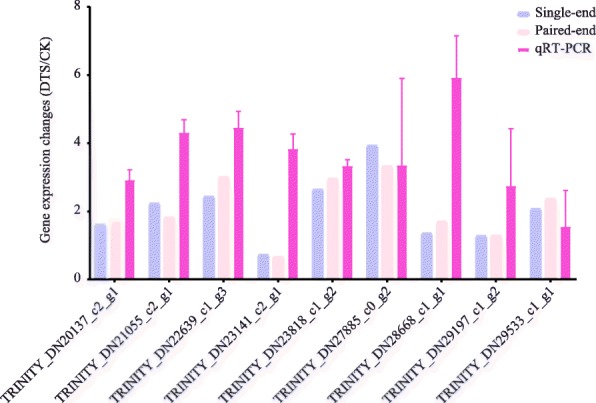
qRT-PCR validation. Gene changes represent the Log2 fold changes of DEGs identified by deep sequencing and the relative normalized expression (2^-ΔΔCt^) identified by qRT-PCR. Error bar represents the standard deviation

We next used qRT-PCR to validate the gene expression levels in sugarcane leaves in response to water deficit. Forward and reverse primers for 9 randomly selected genes and internal control (*actin*) were designed using Primer3 (http://bioinfo.ut.ee/primer3-0.4.0/) and can be accessed in Additional file 5. For each candidate gene, three reactions were performed in each sample (*n* = 3 × 3) and ΔΔCt method was used to present the expression change of a gene in DTS samples compared to CK. As shown in Fig. 6, the expression patterns of all these nine genes identified by qRT-PCR were consistent to those identified by both single-end and paired-end RNA sequencing. Except for TRINITY_DN23141_c2_g1, the other eight genes were up-regulated significantly (Log2FC > 1).

### miRNA regulation of DEGs

To understand the regulation of DEGs by miRNAs, we obtained a total of 412 (261 conserved and 151 novel miRNA) sugarcane miRNAs sequences from a recently published study [38]. Then, psRobot was used to predict the target genes of sugarcane miRNAs in the sugarcane genes assembled in this study. We identified a total of 6577 sugarcane leaf genes that could be regulated by the sugarcane miRNAs. Further, 553 genes were found with differential expression between DTS and CK samples (Additional file 6). These 553 DEGs can be regulated by 175 sugarcane miRNAs, including 89 conserved and 86 novel sugarcane miRNAs. In Table 4, several conserved miRNAs and their target genes were shown, which were dysregulated in sugarcane leaves in response to water deficit. Among them, miR164 that regulates mRNAs encoding NAC domain-containing proteins has been previously reported in sugarcane in response to drought stress [39]. In addition, we identified another three miRNAs (miR172e-3p, miR6225-5p and miR6284) that can regulate the expression of NAC domain-containing protein genes. It is interesting that 7 miRNAs (miR1023a-3p, miR3522, miR6220-5p, miR8014-3p, miR854a, miR854a and miR6220-5p) were identified to target genes encoding disease resistant proteins, which were down-regulated in sugarcane leaves in response to water deficit. It is notable that 8 miRNAs were predicted to regulate WRKY TF genes. Further experiments are required to explore the miRNA regulation in sugarcane leaves and their target gene expression, especially miR6284 and miR854a (Table 4), because they can interact with multiple gene families, such as NAC domain-containing protein, LEA, ethylene-responsive TF, disease resistance protein, ribosomal protein and WRKY TF.

**Table 4:** miRNA regulation of DEGs in DTS and CK samples

miRNA	sequence^a^	target gene	regulation^b^	proteinID	description
miR164a	TGGAGAAGCAGGGCACGTGCA	TRINITY_DN23818_c1_g2	up	NAC22_ARATH	NAC domain-containing protein 21/22
miR172e-3p	GAATCTTTAGACGCATGCAT	TRINITY_DN25766_c0_g2	up		
miR6225-5p	TAACTAGGCTCAAAAGATTCGTCT	TRINITY_DN23398_c1_g2	up	NC100_ARATH	NAC domain-containing protein 100
miR6284	TTTGGACGCATGCATGGAGCATT	TRINITY_DN25766_c0_g2	up	NAC22_ARATH	NAC domain-containing protein 21/22
miR854a	GAGGAGGAAGAGGAGGAGGAG	TRINITY_DN23794_c0_g2	up	NIP21_MAIZE	Aquaporin NIP2–1
miR6220-5p	CTCCATCCCAAATTATAAGACGTT	TRINITY_DN26349_c2_g1	down	TIP42_MAIZE	Aquaporin TIP4–2
miR6248	TAATTTGGAATGGAGGGAGTA				
miR2671j	TTAAAAGATTCGTCTCGTCCA	TRINITY_DN28159_c3_g1	down	GRF5_ORYSJ	Growth-regulating factor 5
miR396e-5p	TCCACAGGCTTTCTTGAACTG				
miR4993	AGCAGCGGCGGTGGCGGATGC	TRINITY_DN22667_c2_g2	up	LEA14_GOSHI	Late embryogenesis abundant protein Lea14-A
miR5175a	TTAGAATTTGGAAAGGAGGGA	TRINITY_DN26424_c6_g1	up	LEA5_CITSI	Late embryogenesis abundant protein Lea5
miR5205b	CTTATAATTTGGGATGGAGGGAGT				
miR5568f-3p	TCTTATAATTTGGAATGGAGG				
miR6220-3p	AATGACTTATAATTTGGGATGGAG				
miR6248	TAATTTGGAATGGAGGGAGTA				
miR5568f-3p	TCTTATAATTTGGAATGGAGG	TRINITY_DN16664_c1_g1	up	LEA5D_GOSHI	Late embryogenesis abundant protein Lea5-D
miR6248	TAATTTGGAATGGAGGGAGTA				
		TRINITY_DN17990_c0_g1	up	EF114_ARATH	Ethylene-responsive transcription factor ERF114
miR854a	GAGGAGGAAGAGGAGGAGGAG	TRINITY_DN20050_c2_g1	up		
		TRINITY_DN24075_c2_g3	up	ERF53_ARATH	Ethylene-responsive transcription factor ERF053
miR4993	AGCAGCGGCGGTGGCGGATGC	TRINITY_DN22160_c1_g2	up	ERF60_ARATH	Ethylene-responsive transcription factor ERF060
miR1023a-3p	CAAGAATTGGATGAAGTGCAT	TRINITY_DN22589_c0_g1	down	R13L3_ARATH	Putative disease resistance RPP13-like protein 3
miR3522	AGCCCAAGTCGAGACAGCTGA	TRINITY_DN20821_c1_g2	down		
miR6220-5p	CTCCATCCCAAATTATAAGACGTT	TRINITY_DN17917_c0_g2	down	RGA1_SOLBU	Putative disease resistance protein RGA1
miR8014-3p	ATTAATCAATGTTTGGACAATT	TRINITY_DN20066_c3_g1	down	RGA3_SOLBU	Putative disease resistance protein RGA3
miR854a	GAGGAGGAAGAGGAGGAGGAG	TRINITY_DN22688_c0_g1	down		
		TRINITY_DN25829_c1_g3	up		
miR6220-5p	CTCCATCCCAAATTATAAGACGTT	TRINITY_DN25593_c2_g1	up	RGA4_SOLBU	Putative disease resistance protein RGA4
miR5205b	CTTATAATTTGGGATGGAGGGAGT	TRINITY_DN24732_c1_g1	down	RK12_ORYSJ	50S ribosomal protein L12, chloroplastic
miR5568f-3p	TCTTATAATTTGGAATGGAGG				
miR6248	TAATTTGGAATGGAGGGAGTA				
miR854a	GAGGAGGAAGAGGAGGAGGAG	TRINITY_DN18780_c1_g2	down	RK28_ARATH	50S ribosomal protein L28, chloroplastic
		TRINITY_DN25639_c1_g1	down	WRK19_ARATH	Probable WRKY transcription factor 19
		TRINITY_DN23859_c0_g1	up	WRK40_ARATH	Probable WRKY transcription factor 40
miR172e-3p	GAATCTTTAGACGCATGCAT	TRINITY_DN19490_c0_g1	up	WRK54_ARATH	Probable WRKY transcription factor 54
miR6284	TTTGGACGCATGCATGGAGCATT				
miR1436	ACTTTCAATGGGACGGAGGGAGT	TRINITY_DN22501_c1_g1	up	WRK57_ARATH	Probable WRKY transcription factor 57
miR4993	AGCAGCGGCGGTGGCGGATGC				
miR5205b	CTTATAATTTGGGATGGAGGGAGT				
miR6220-3p	AATGACTTATAATTTGGGATGGAG				
miR5139	AACCTGGCTCCGATACCA	TRINITY_DN18434_c1_g4	up	5NG4_PINTA	Auxin-induced protein 5NG4

^a^miRNA sequence

^b^up−/down-regulation of the gene in DTS compared to CK samples

## Discussion

In this study, we used paired-end and single-end sequencing technologies to investigate the gene expression profiles of sugarcane leaves in response to water deficit. This is the first transcriptome study of *Saccharum narenga* species using the deep sequencing technology. We identified 104,644 sugarcane leaf genes (N50 = 1605 bp), of which 21.40% can be aligned to *Sorghum bicolor*. In DTS and CK samples we identified 30,297 genes (> 5 TPM), of which single-end sequencing identified 4551 (2327 up-regulated and 2224 down-regulated) DEGs while paired-end sequencing identified 4212 (2268 up-regulated and 1944 down-regulated) DEGs in DTS samples, compared to CK. Functional analysis showed that DEGs in DTS and CK were involved in “circadian rhythm – plant” and “plant hormone signal transduction”. Our further analysis of gene expression patterns revealed that several gene families, such as aquaporin, LEA, auxin related protein, TF, heat shock protein and LHCB, might be involved in *Saccharum narenga*leaves in response to water deficit.

In plant, the perception of water deficit can trigger the activation of abscisic acid (ABA)-dependent and ABA-independent regulatory systems that govern drought-inducible gene expression [2]. Genes in response to the water-deficit stress can be divided into two groups based on their functions [14, 15]. The first group of gene products are involved in the protection of cells and in the regulation of signal transduction pathways of stress responses, such as chaperons, LEA proteins, water channel proteins, heat shock proteins and lipid-transfer proteins. In this study, 90 genes encoding different chaperons have been identified (Additional file 2) in *Saccharum narenga*leaves. Differential expression analysis identified 14 up-regulated and 7 down-regulated chaperone genes in DTS samples compared to CK (Additional file 4). These chaperones may function in the protection of proteins from degradation and the action of proteinases in sugarcane leaves under drought stress [40]. LEA genes have been reported to be involved in the protection of cell structures from the effects of water loss [41]. The LEA gene products are proposed to be located in cytoplasm and have several features such as hydrophilism, biased in amino acid composition, and lacking in Cys and Trp [41]. They mainly function in sequestration of ions, protection of other proteins or membranes, and renaturation of unfolded proteins [41]. In this study, five LEA genes are identified to be up-regulated in sugarcane leaves in response to water deficit, which is consistent with other plant species like Arabidopsis [42], rice [6], maritime pine [10], loranthus [12] and sugarcane cultivars [11].

Genes encoding heat shock proteins have also been identified to contribute to drought-stress tolerance in plants [2]. In different tissues (root, stem and leaf) of an Indian sugarcane variety (CoS 767), genes encoding heat shock proteins are shown to be up-regulated in response water deficit [18]. In addition, genes encoding ribosomal proteins and putative disease-resistance proteins are also identified to be up-regulated in these sugarcane tissues in response to water deficit [18]. In this study, we confirmed their dysregulation in sugarcane leaves in response to water deficit. Further, we found not only up-regulated but also down-regulated genes that can encode ribosomal proteins and putative disease resistance proteins. For example, genes encoding 30S and 50S ribosomal proteins were up-regulated while genes encoding 60S ribosomal proteins were down-regulated in DTS samples compared to CK (Fig. 5g). DEGs identified in this study include 14 up-regulated and 45 down-regulated genes encoding disease resistance proteins (Additional file 4).

Like ribosomal protein and disease resistance protein, it is difficult to determine the regulation of genes encoding the major water channel proteins – aquaporins – in plant leaves under water-deficit stress. Aquaporins are water transporter proteins that play an important role in adjusting the water status in response to environmental changes [43]. In sugarcane, only TIP aquaporin genes have been identified to be induced by water-deficit stress [17]. Interestingly, our results revealed that the TIP aquaporin genes were down-regulated in *Saccharum narenga*leaves in response to water deficit while genes encoding NIP and PIP aquaporins were up-regulated. It has been shown that in Arabidopsis the loss of TIP aquaporin leads to cell and plant death [44]. The functions of different aquaporin families in *Saccharum narenga*leaves in response to water deficit require to be explored with further experiments. Other gene products involved in signal transduction like lipid-transfer proteins and LHCB proteins have also been reported to be induced by the water-deficit stress in commercial sugarcane varieties [1, 17].

The second group of gene products in response to water deficit in plants is comprised of regulatory proteins that are involved in further regulation of signal transduction and stress-responsive gene expression, such as various TFs and dehydration-responsive element [2]. Rocha and colleagues identified 93 TF genes, including MYB and WRKY TFs, differentially expressed in sugarcane plant (cultivar SP90–1638) in response to water deficit [45]. In our study, ABA-inducible TF genes (e.g., MYB and NAC) and ABA-independent TF genes (e.g., ERF) were identified to be differentially expressed in *Saccharum narenga*leaves in response to water deficit (Fig. 5d, Additional file 4). In addition, genes encoding DIVARICATA, heat stress, TCP TFs and nuclear transcription factor Y subunit were also identified, for the first time, to be differentially expressed in the leaves in response to water deficit (Fig. 5d, Additional file 4). The up-regulation of heat stress TFs might be responsible for the increase of heat shock proteins in DTS samples. The dysregulation of these TF genes requires further experiments to understand their regulation in *Saccharum narenga*leaves in response to water deficit. In addition, another group of ABA-independent proteins, dehydration-responsive element-binding proteins, were up-regulated in DTS samples compared to CK, which is consistent with other studies about sugarcane plants under drought stress [11, 17]. Gentile and colleagues reviewed and uncovered a complex regulation of sugarcane miRNAs in response to drought [39]. They point out that the cultivar, the growth conditions and the duration of stress have influence on the observation of miRNA expression profiles. Some miRNAs have been reported to be dysregulated in sugarcane exposed to drought, such as miR160, miR166, miR169, miR171 and miR399 [39]. It has been found in one sugarcane cultivar that the down-regulation of miR169 could increase the expression of glutathione S-transferase (GST) therefore reduce the toxic effects of reactive oxygen species [39]. Although this regulation was not observed in this study, the down-regulation of genes encoding C7254_GLYUR (11-oxo-beta-amyrin 30-oxidase) and ACA6_ORYSJ (Probable calcium-transporting ATPase 6, plasma membrane-type) might be regulated by miR169 in the sugarcane leaves. In addition, we found that some miRNAs could target the DEGs that have been described as being related to drought stress and/or increasing tolerance to water deficit, such as miR164 targeting NAC domain-containing TFs [46]. In addition, we found two miRNAs miR6284 and miR854a of interest because they can regulate multiple gene families, such as NAC domain-containing protein, LEA, ethylene-responsive TF, disease resistance protein, ribosomal protein and WRKY TF. miR854a has been reported to be induced by drought stress in other plants, including rice [47], maize [48], banana [8] and tea [13]. miR6284 has been reported to be an arbuscular mycorrhiza (AM)-responsive miRNA in tomato [49]. The potential regulation of these two miRNAs and their target genes indicates they might be functional in sugarcane leaves in response to water deficit.

Our study agreed the expression changes of some known gene families in *Saccharum narenga* under water stress, such as LEA, ribosomal protein, heat shock protein, and some TFs. However, it wa shown that aquaporin and DIVARICATA, heat stress, TCP TFs and nuclear transcription factor Y subunit require more experiments to explore their functions in the plants in response to water loss. More importantly, deep sequencing and de novo assembly analysis enabled us to identify new genes in the process. In total, we identified that 3389 genes were differentially expressed in DTS compared to CK, and that 2232 (65.86%) were similar to known gene families (Additional file 4). Some of them are probably specific to this species or possible noncoding genes (Fig. 2), and some of them were highly expressed (> 100 TPM) and dysregulated in DTS, such as TRINITY_DN29490_c3_g1, TRINITY_DN15129_c0_g1, TRINITY_DN25403_c0_g5 and TRINITY_DN22079_c0_g3 (Additional file 4). The reason why these possible novel genes were identified require more experiments to explore. Further, their functions in drought tolerance in *Saccharum narenga* are still unknown and need to be studied.

## Conclusions

In conclusion, we used deep sequencing technology to investigate the transcriptome profiles in *Saccharum narenga,* a wild type sugarcane leaves in response to water deficit. Among the assembled 104,644 *Saccharum narenga* genes, 38,721 can be annotated to other databases, such as NR, UniProt, KEGG and GO. In DTS and CK samples we identified 30,297 genes whose expression levels were greater than 5 TPM. Then, edgeR was used for differential expression analysis and 3389 DEGs (1772 up-regulated and 1617 down-regulated) were consistent in paired-end and single-end sequencing. Functional analysis showed they were involved in pathways like “response to blue light”, “metabolic pathways” and “plant hormone signal transduction”. We observed the expression patterns of several gene families in sugarcane leaves under the water-deficit stress, such as aquaporin, LEA, auxin related proteins, TF, heat shock protein, LHCB, disease resistance protein and ribosomal proteins. Interestingly, genes encoding NIP and PIP aquaporins were up-regulated in *Saccharum narenga* leaves in response to the water-deficit stress while genes encoding TIP aquaporins were down-regulated. We also found that genes encoding 30S and 50S ribosomal proteins were up-regulated while 60S ribosomal protein genes were down-regulated in DTS samples compared to CK. Further, we showed that the DEGs identified in this study might be regulated by miRNAs. This is the first sugarcane leaf transcriptome study using both single-end and paired-end sequencing technologies. Genes encoding LHCB, DIVARICATA and heat stress TFs were first reported, to our knowledge, in sugarcane leaves under water stress. Our findings will improve the understanding of the mechanism gene regulation in sugarcane leaves under drought stress. The output of this study will also contribute to the sugarcane breeding program.

## Additional files

Additional file 1: Gene Ontology annotation for the assembled sugarcane transcriptome. (PDF 192 kb)

Additional file 2: Gene expression profile in all samples (> 5 TPM). (XLSX 2393 kb)

Additional file 3: Differential expression analysis of all genes in DTS samples compared to CK. (XLSX 12539 kb)

Additional file 4: DEGs consistent in paired-end and single-end sequencing technologies. (XLSX 649 kb)

Additional file 5: qPCR primers. (XLSX 9 kb)

Additional file 6: miRNA regulation of the DEGs identified in DTS and CK samples. (XLSX 61 kb)

## Abbreviations

CK: Control sugarcane leaves
COG: Clusters of Orthologous Groups
DTS: Drought treated sugarcane leaves
ERF: Ethylene-responsive transcription factor
GO: Gene ontology
GST: Glutathione S-transferase
HSP: Heat shock protein
KEGG: Kyoto encyclopedia of genes and genomes
KOG: EuKaryotic Orthologous Group
Log2FC: Log2 fold change
NOG: Non-supervised orthologous group
NR: NCBI non-redundant database
RSEM: RNA-Seq by Expectation-Maximization
TF: Transcription factor
TPM: Transcripts per million reads
