# Supplementary figures and images for: Comprehensive transcriptome analysis reveals genes in response to water deficit in the leaves of Saccharum narenga (Nees ex Steud.) hack
Source: BMC Plant Biol. 2018 Oct 20;18:250. doi: 10.1186/s12870-018-1428-9 (PMC6195978; doi:10.1186/s12870-018-1428-9)

Gene Ontology annotation of the assembled sugarcane genes

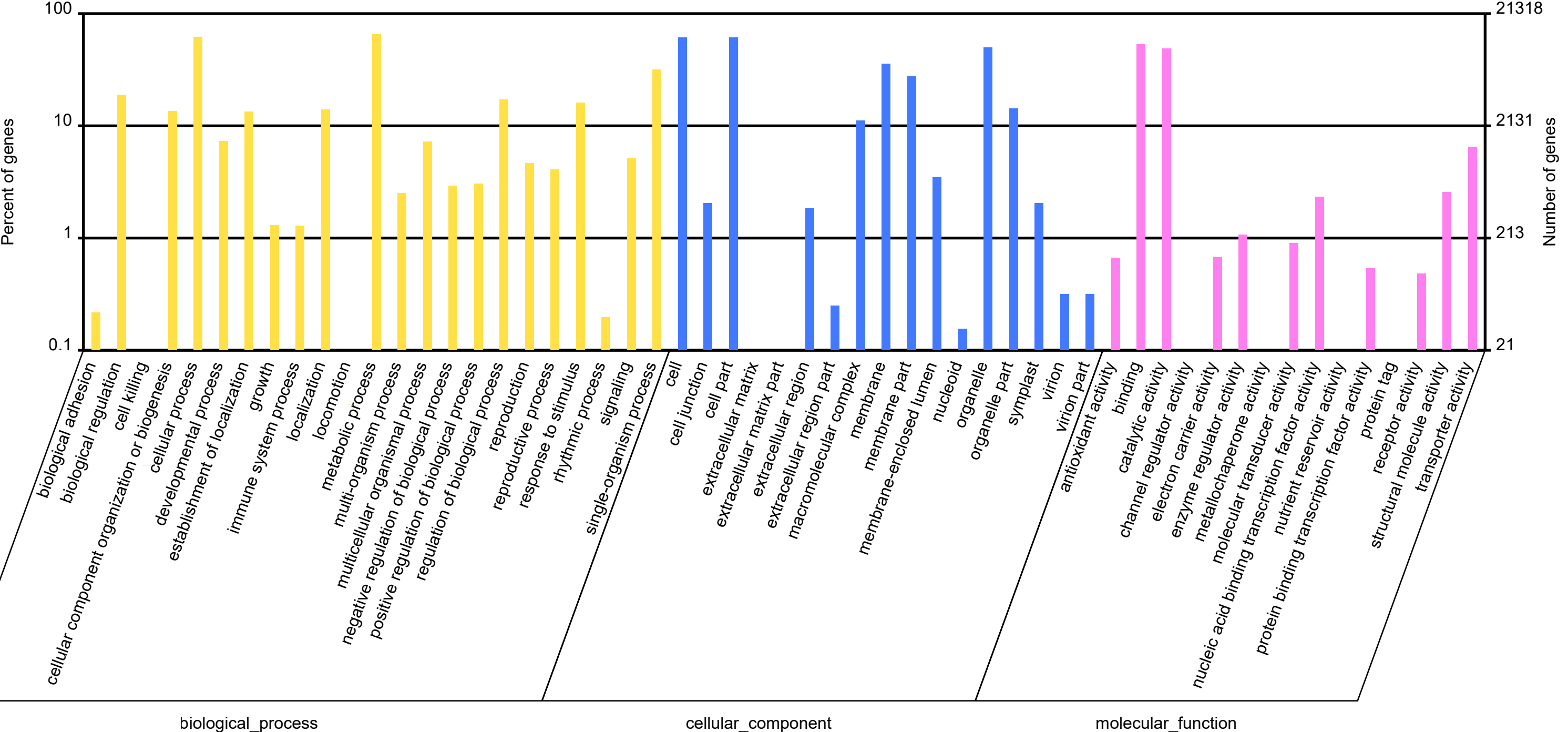

Supplement: Supplementary file 1 — Gene Ontology annotation for the assembled sugarcane transcriptome. (PDF 192 kb) [file 12870_2018_1428_MOESM1_ESM.pdf]
